# O aborto legal em casos de gravidez decorrente de violência sexual: percepções e vivências de médicas e médicos obstetras

**DOI:** 10.1590/0102-311XPT124423

**Published:** 2024-06-14

**Authors:** Ana Júlia Saldanha Lehnen, Mariana Rabello, Geiza Martins Barros, Fernanda Freitas Oliveira Cardoso

**Affiliations:** 1 Maternidade Escola, Universidade Federal do Rio de Janeiro, Rio de Janeiro, Brasil.; 2 Fundação Oswaldo Cruz, Rio de Janeiro, Brasil.

**Keywords:** Aborto Legal, Violência Sexual, Assistência Perinatal, Médicos, Legal Abortion, Sexual Violence, Perinatal Care, Physicians, Aborto Legal, Violencia Sexual, Atención Perinatal, Médicos

## Abstract

Este estudo objetivou analisar as percepções de obstetras e residentes de ginecologia-obstetrícia, atuantes numa maternidade escola federal, sobre o aborto legal em casos de gravidez decorrente de violência sexual, desvelando suas motivações, resistências e sentimentos, e identificando suas experiências com o tema. A primeira etapa correspondeu ao preenchimento de um questionário autoaplicável. Os critérios de seleção foram: obstetras vinculados ao centro obstétrico; diretor da divisão médica; e residentes do programa de ginecologia-obstetrícia da instituição. Obtiveram-se 36 questionários respondidos. A segunda etapa correspondeu à realização de uma entrevista, tendo sido utilizado o critério de amostragem por saturação e foram entrevistados seis médicos. As entrevistas foram analisadas pelo método de análise de conteúdo, na modalidade temática. Os questionários retrataram que todos os participantes já haviam prestado assistência a mulheres em situação de violência sexual e que a maioria já havia participado da realização de um aborto legal. As entrevistas evidenciaram os dilemas enfrentados pelos profissionais na assistência a esses casos e a escassez da formação profissional em relação à temática. A palavra da mulher foi tida ora como objeto de suspeição em relação à veracidade do estupro, ora como capaz de suscitar afetação das profissionais em suas escutas, o que possibilitou que essas se aproximassem das vítimas e ofertassem uma assistência mais humanizada. Os resultados apontaram para a importância da temática ser abordada nos campos da saúde e da formação para além do enfoque técnico-científico, visando produzir novas estratégias de cuidado.

## Abstract (en)

This study aimed to analyze the perceptions of obstetricians and gynecology-obstetrics residents at a federal school maternity hospital regarding legal abortion in cases of sexual violence pregnancy, understand their motivations, strengths, and feelings, and identify their experience with this topic. The first stage consisted of answering a self-administered questionnaire. The selection criteria were: obstetricians linked to the obstetric center, director of the medical division, and residents of the institution’s obstetrics-gynecology program. In total, 36 questionnaires were answered and returned. The second stage corresponded to an interview using a saturation sampling criterion. Six physicians were interviewed. The interviews were evaluated using a thematic content analysis. The questionnaires showed that all participants had already provided care to women in situations of sexual violence and that most of them had already participated in a legal abortion procedure in these cases. The interviews highlighted the dilemmas faced by professionals in providing care to these women and the lack of professional training to handle these cases. The speeches of women were sometimes seen as an object of suspicion regarding the veracity of sexual violence, and sometimes as an object that caused professionals to feel emotionally affected while listening to them, allowing professionals to approach the victims and offer more humanized care. The results pointed to the importance of addressing this topic in the areas of health and providing training beyond the technical-scientific focus in order to support the development of new care strategies.

## Resumen

El estudio tuvo como objetivo analizar las percepciones de obstetras y residentes de gineco-obstetricia de una maternidad escolar federal sobre el aborto legal en casos de embarazo resultante de violencia sexual, revelar sus motivaciones, resistencias y sentimientos, e identificar sus experiencias con el tema. La primera etapa consistió en completar un cuestionario autoadministrado. Los criterios de selección fueron los siguientes: obstetras vinculados al centro obstétrico; director de la división médica; y residentes del programa de gineco-obstetricia de la institución. Se obtuvieron 36 cuestionarios cumplimentados. La segunda etapa consistió en una entrevista, utilizando un criterio de muestreo por saturación. Se entrevistó a 6 médicos. Las entrevistas se analizaron mediante el método de análisis de contenido, en la modalidad temática. Los cuestionarios mostraron que todos los participantes ya habían brindado asistencia a mujeres en situación de violencia sexual y que la mayoría ya había participado en la realización de un aborto legal en estos casos. Las entrevistas pusieron de manifiesto los dilemas que enfrentan los profesionales en la asistencia a estos casos y la escasa formación profesional con relación al tema. La palabra de la mujer fue vista a veces como objeto de sospecha con respecto a la veracidad de la violación, y a veces como un objeto capaz de suscitar la afectación de las profesionales en sus escuchas, lo que les permitió acercarse a las víctimas y ofrecer una asistencia más humanizada. Los resultados señalaron la importancia de que la temática sea abordada en los campos de la salud y de la formación más allá del enfoque técnico-científico, con el objetivo de producir nuevas estrategias de cuidado.

## Introdução

O aborto induzido nos casos de gravidez decorrente de violência sexual tem previsão legal no Brasil desde 1940, conforme *Decreto-Lei nº 2.848* ^1^, de 7 dezembro de 1940, artigo 128, inciso II do Código Penal brasileiro. Apesar das diretrizes da Organização Mundial da Saúde (OMS) ^2^ não caracterizarem tal prática enquanto um ato exclusivamente médico, ela é restrita à essa categoria no Brasil, embora seja necessária a avaliação de uma equipe multidisciplinar para a sua realização quando a gravidez decorre de estupro ^3^. Entretanto, apesar do âmbito da saúde ser o responsável pela realização do aborto nesses casos, o primeiro serviço de saúde que ofertava esse tipo de assistência foi criado apenas em 1989 ^4^.

Em 1999, o Ministério da Saúde publicou, pela primeira vez, a norma técnica sobre *Prevenção e Tratamento dos Agravos Resultantes da Violência Sexual Contra Mulheres e Adolescentes* ^5^, seguida da norma técnica sobre *Atenção Humanizada ao Abortamento* ^6^, em 2005. 

Elas representaram marcos importantes em relação ao amparo para mulheres vítimas de violência sexual e ao aborto legal, por serem documentos que visam auxiliar os serviços e os profissionais de saúde na organização e desenvolvimento de uma assistência qualificada e eficaz ^3^ ^,^ ^4^.

No cenário atual, todavia, constata-se que os marcos legais e a instituição de unidades de referência para assistência ao aborto legal não garantem a efetivação do direito das mulheres vítimas de violência sexual ^3^ ^,^ ^4^. Entre os principais problemas relacionados à assistência a esses casos, cabe citar a carência de recursos humanos para cuidar do conjunto de necessidades das vítimas, a insuficiência de serviços para atender a demanda e a pouca divulgação desses já existentes ^7^. Além disso, dentro dos serviços que prestam esse tipo de assistência, ainda existem barreiras por parte dos profissionais que, por vezes, advém de questões morais e religiosas, o que vai na contramão do que é preconizado pelas normas técnicas do Ministério da Saúde ^4^ ^,^ ^7^ ^,^ ^8^.

Quando pensamos a respeito dos profissionais de saúde inseridos nesse contexto, percebemos que o entendimento da relação entre a temática da violência com o campo da saúde ainda é recente, o que a torna distante da formação e da prática de muitos profissionais. Essas lacunas acabam repercutindo na visão deles acerca dos casos de violência sexual, e de como entendem sua prática profissional nesse tipo de assistência. Soma-se a isso, ainda, o contexto sociocultural do Brasil e questões de ordem moral, ética e religiosa existentes quanto à prática do aborto, criminalizada e estigmatizada no país ^3^ ^,^ ^7^.

Dessa forma, os profissionais de saúde envolvidos na assistência ao aborto legal de mulheres vítimas de violência sexual podem experienciar diversos desconfortos, como sentimentos e reações intensas que podem se entrelaçar a questões de ordem pessoal. Tais reações e sentimentos surgem, por vezes, como fruto da sensibilização perante a aflição da mulher atendida. Alguns profissionais se referem a uma motivação altruísta ao se envolver com esses casos, visando minimizar o sofrimento da mulher, o que por vezes passa por histórias pessoais ou de pessoas próximas relacionadas à temática da violência e do aborto ^7^ ^,^ ^8^.

Em outros casos, as reações e sentimentos experimentados pelos profissionais podem surgir a partir dos conflitos e dos dilemas morais e éticos que emergem diante da possibilidade da realização de um aborto legal. As questões religiosas, por vezes, ocupam lugar central na trama desses dilemas, principalmente quando há a concepção de que o aborto é um pecado, sobre o qual a culpa incide tanto na mulher quanto no profissional envolvido. A cultura profissional e os avanços tecnológicos do campo da medicina fetal são fatores que também podem compor os conflitos de alguns profissionais com o aborto legal. Ainda, o receio em relação ao julgamento da sociedade e o medo de responder criminalmente pela realização do procedimento, bem como da gravidez não ter sido fruto de estupro, podem se somar aos fatores que compõe tais conflitos ^7^ ^,^ ^8^ ^,^ ^9^.

Tendo em vista o exposto, este estudo se propôs a ouvir os profissionais de saúde responsáveis pela realização do aborto legal nesses casos - os médicos. O objetivo da pesquisa foi analisar as percepções de médicos obstetras e residentes de obstetrícia vinculados ao centro obstétrico de uma maternidade escola federal em relação ao aborto legal, realizado quando a gravidez é decorrente de violência sexual, bem como desvelar suas motivações, resistências e sentimentos, identificando suas experiências com o tema.

## Materiais e método

Esta pesquisa foi feita em uma maternidade escola federal referência em medicina fetal, que conta com cerca de 20 obstetras no centro obstétrico e 33 residentes de programas de ginecologia-obstetrícia. A pesquisa foi realizada entre agosto e novembro de 2022, após aprovação no Comitê de Ética em Pesquisa da Maternidade Escola da Universidade Federal do Rio de Janeiro (parecer nº 5.524.753, em 13 de julho de 2022). Trata-se de um estudo descritivo e de abordagem qualitativa, que ocorreu em duas etapas e buscou responder à seguinte questão norteadora: “Quais as percepções dos médicos em relação ao aborto legal por gestação decorrente de violência sexual?”.

A primeira etapa correspondeu à descrição do perfil pessoal e profissional dos participantes, bem como da experiência desses com o tema, por meio do preenchimento de um questionário autoaplicável, com perguntas fechadas. Os critérios de seleção foram: obstetras vinculados ao centro obstétrico da instituição; diretor da divisão médica; e residentes dos programas de ginecologia-obstetrícia vinculados à instituição. Os participantes foram convidados a participar do estudo no local onde desenvolvem suas atividades, em momento oportuno, e assinaram Termo de Consentimento Livre e Esclarecido (TCLE) específico para essa etapa. Foram obtidos 36 questionários respondidos - 12 de obstetras da instituição e 24 de residentes. As respostas foram tabuladas em planilha do programa Microsoft Excel (https://products.office.com/) e os dados foram analisados de forma a descrever o perfil da amostra em relação às questões abordadas.

A segunda etapa correspondeu a uma entrevista realizada pela autora principal do estudo (A.J.S.L.), que seguiu um roteiro semiestruturado. Para a escolha dos participantes dessa etapa, atentou-se a sexo, posição na instituição e ter declarado ou não restrições quanto a realização do aborto legal em casos de gravidez decorrente de estupro, tendo sido preconizada uma variedade quanto a esses aspectos na amostra.

Para definição do número de participantes dessa etapa, foi aplicado o critério de amostragem por saturação, ferramenta frequentemente utilizada em pesquisas qualitativas de diferentes áreas, inclusive no campo da saúde. Tal técnica é utilizada para definir o número da amostra, com a suspensão da inclusão de novos participantes a partir do momento em que os dados obtidos passam a apresentar redundância ou repetição na visão do pesquisador. Essa avaliação é feita a partir de um processo contínuo de análise dos dados obtidos desde o início da coleta ^10^.

Foram entrevistados 6 médicos, 3 do corpo médico da instituição e 3 do programa de residência de ginecologia-obstetrícia. As entrevistas foram realizadas após assinatura de TCLE específico a essa etapa. A maioria ocorreu na própria maternidade, exceto por uma que foi realizada *online*, em uma sala no Google Meets (https://meet.google.com/), por conveniência do entrevistado. O tempo de duração variou entre 7 e 28 minutos, e todas foram gravadas após consentimento dos participantes. Posteriormente, foram transcritas e analisadas por meio do método de análise de conteúdo, conforme postulado por Bardin ^11^, na modalidade temática.

A análise de conteúdo se organiza em 3 etapas: pré-análise; exploração do material; e tratamento dos resultados, inferência e interpretação ^11^. Neste estudo, a primeira etapa da análise correspondeu à leitura flutuante das transcrições das entrevistas, a partir das quais foi possível se deixar invadir por impressões e orientações iniciais, levando à construção de categorias. Na segunda etapa, as falas e percepções dos médicos foram classificadas a partir das categorias construídas na etapa anterior, agrupadas pelos núcleos de sentido. Já na última etapa, foram realizadas as análises e articulações com o referencial teórico utilizado para o estudo.

## Resultados e discussão

A partir da análise dos questionários, verificou-se que a idade dos participantes variou entre 25 e 56 anos. A maioria era mulher (83,3%), se autodeclarou de raça/cor branca (83,3%) e referiu estar solteira (52,7%). Em relação à orientação religiosa, 52,7% se declararam católicos/cristãos, 25% se declararam espíritas, 19,4% não tinham orientação religiosa ou se declararam ateus e 2,7% referiram orientação religiosa de matriz africana (Tabela 1). Dos 29 que manifestaram alguma orientação religiosa, 9 se declararam como praticantes.

**Tabela 1:** Perfil dos participantes.

Variáveis	n	%
Idade (anos)		
< 30	23	63,89
30-40	7	19,44
41-50	4	11,11
> 50	2	5,56
Sexo		
Mulher	30	83,33
Homem	6	16,67
Raça/Cor		
Branca	30	83,33
Parda	5	13,89
Ignorada	1	2,78
Situação conjugal		
Solteiro	19	52,78
Em relação estável/Casado	17	47,22
Orientação religiosa		
Católica/Cristã	19	52,78
Espírita	9	25,00
Sem orientação religiosa/Ateu	7	19,44
Candomblé	1	2,78
Tempo de formação		
Formado na graduação há < 1 ano	3	8,33
Formado na graduação há 1-10 anos	22	61,11
Formado na graduação há > 10 anos	11	30,56
Tempo de atuação na instituição (anos)		
< 1	11	30,56
1-10	20	55,56
> 10	4	11,11
Ignorado	1	2,78

O tempo de formação dos participantes variou entre 7 meses e 35 anos, e o tempo de atuação na instituição em que a pesquisa foi realizada variou entre 5 meses e 20 anos. Todos os participantes referiram já terem prestado assistência a mulheres em situação de violência sexual. A maioria (88,8%) afirmou já ter realizado ou estado envolvido diretamente na realização de um aborto legal por gravidez decorrente de violência sexual.

Em relação à formação e capacitação, 21 participantes apontaram terem tido um contato breve ou de forma que consideram insuficiente com o tema durante a formação profissional, 10 referiram que o tema foi explorado de forma que consideram satisfatória e 5 responderam que não tiveram contato com o tema na formação profissional até o momento. Dos 36 participantes, apenas um sinalizou já ter participado de alguma prática educativa a respeito do tema na instituição em que foi realizada a pesquisa. Pouco mais da metade (55,5%) se consideram capacitados para prestarem assistência à situação de violência sexual e ao aborto legal, 41,6% responderam que se consideram parcialmente capacitados e 2,7% apontaram que não se consideram capacitados.

Quanto à segunda etapa, foram entrevistados 4 mulheres e 2 homens. Três mulheres eram residentes e uma era obstetra da instituição, assim como os homens entrevistados. A idade variou entre 26 e 51 anos e todos se declararam brancos. Dois se declararam católicos, 2 espíritas, e 2 sem orientação religiosa. Dois entrevistados afirmaram já terem se recusado a realizar o procedimento de aborto legal em casos de gravidez decorrente de violência sexual.

A seguir, serão apresentadas as categorias que emergiram da análise das entrevistas.

### O dever e o fazer médico: possibilidades e limitações pessoais

Apareceu de forma unânime nas entrevistas o entendimento da assistência ao aborto legal em casos de gravidez decorrente de violência sexual como algo que faz parte da função do médico, estando incumbido em seu dever profissional. A assistência à saúde da mulher e a garantia da efetivação do direito ao aborto legal para as vítimas de violência sexual apareceram relacionadas a essa função e dever. Os protocolos institucionais e a legislação do país foram apresentados como norteadores essenciais da prática profissional nessas situações.

Entretanto, apesar do entendimento da assistência a esses casos enquanto algo que faz parte da função e do dever do médico, foi nítido na fala dos entrevistados o quanto as questões pessoais dos profissionais se relacionam a isso, gerando dilemas para alguns deles. Nas entrevistas realizadas, as questões de ordem moral e religiosa foram as consideradas relevantes na percepção dos entrevistados.

Dois entrevistados, ambos do corpo médico da instituição, apresentaram de forma explícita o quanto as suas crenças religiosas e pessoais assomavam questões quanto à prática do aborto legal. Em um desses casos, a entrevistada abordou os dilemas que enfrenta ao se deparar com essas situações na assistência, apesar de não se abster de prestar atendimento nesses casos:

“*Eu comecei a questionar isso* (...) *Se eu tava fazendo uma coisa contrária aos desígnios da religião* (...) *E aí acabei entrando nessa questão assim, profissão* versus *religião… Mas eu sou muito realizada profissionalmente, então… Pra mim não era uma hipótese me afastar da profissão… É me adaptar ao que eu tenho que fazer na minha profissão*” (Participante 1).

Outro médico alegou ter dificuldade no “*prosseguimento do aborto legal*” (Participante 2) por questões pessoais e religiosas, relacionadas ao entendimento da existência de uma vida desde o momento da concepção. Ele trouxe que não se abstém de atuar quando há complicações relacionadas ao procedimento ou quando é necessário realizar esvaziamento uterino após a eliminação do feto, mas que, enquanto houver “*embrião ou feto em viabilidade*” (Participante 2), conta com que outra pessoa da equipe tome frente da situação.

“*Eu tenho muita dificuldade… Eu como profissional, de ser a pessoa que vai intervir nessas situações.* (...) *Eu acho que a gente não deve pensar nunca inicialmente na gente e depois na paciente… Mas como nós somos uma equipe, normalmente as pessoas se distribuem nas suas atribuições de acordo com aquilo que acredita*” (Participante 2).

É oportuno destacar que a Participante 1 declarou não ser praticante de sua orientação religiosa. Dessa forma, percebe-se que, mesmo entre os profissionais que não se declaram praticantes, as crenças de cada um ainda podem se configurar como relevantes na trama dos dilemas vivenciados por eles.

As formas como as equipes se relacionam entre si para prestarem assistência a esses casos também apareceu de forma preponderante nas demais entrevistas. Foi abordado que esse é um tema bastante discutido dentro dos plantões, e que os profissionais apresentavam suas possibilidades e limitações em relação à realização do aborto legal nesses casos. Em algumas entrevistas, entretanto, esse diálogo foi caracterizado como uma discussão que gera certo desgaste emocional e psicológico dentro das equipes, por haver divergências culturais e religiosas importantes entre os profissionais.

Tais achados vão ao encontro do estudo de Farias & Cavalcanti ^12^, que também identificou questões religiosas e relacionadas ao valor da vida como fatores que contribuem para que os profissionais envolvidos na assistência ao aborto legal se deparem com dilemas em as suas funções profissionais. Outro estudo, realizado com 1.174 estudantes de medicina, encontrou uma relação significativa entre religiosidade e objeção de consciência ^13^.

A objeção de consciência é abordada no Código de Ética Médica ^14^ como um direito do médico não praticar condutas que estejam em desacordo com seus valores individuais. No que diz respeito aos casos de aborto legal, a legislação expõe que esse dispositivo não pode anular o direito de acesso da mulher a esse tipo de assistência, sendo de responsabilidade da instituição de saúde garantir a presença de profissionais que não apresentem restrições quanto à realização do procedimento, e sendo vedado ao médico se recusar a prestar assistência ao abortamento em casos de urgência ou emergência, na ausência de outro médico que o faça ou quando sua recusa implique danos à saúde da paciente ^3^ ^,^ ^15^.

Diniz ^16^ ^,^ ^17^ nos provoca a pensar o dispositivo da objeção de consciência não enquanto um direito fundamental do profissional, mas como uma forma de proteção a um sentimento. Ela postula que o entendimento dele como um direito universal e absoluto ameaça as necessidades da população, podendo levar a uma desestabilização do sistema de saúde pelo risco permanente de recusa de assistência por parte dos profissionais.

Em contrapartida, o entendimento da objeção de consciência enquanto um dispositivo de proteção aos sentimentos dos profissionais aponta para a importância de organizar o encontro entre os dogmas e os sentimentos deles com as necessidades de saúde das mulheres vítimas de violência sexual, que buscam a efetivação do direito ao aborto legal em um Estado laico. Por essa perspectiva, pode-se pensar em estratégias, como medidas administrativas de acomodação interna dos serviços de saúde e arranjos institucionais das equipes, que visem escutar e acolher o sofrimento e as angústias do profissional que declara objeção de consciência, sem negligenciar a assistência à mulher vítima de violência sexual ^16^ ^,^ ^17^.

### O acesso ao aborto legal na rede de atenção: impressões sobre os serviços de saúde

A Rede de Atenção à Saúde se caracteriza como “*arranjos organizativos de ações e serviços de saúde, de diferentes densidades tecnológicas, que integradas por meio de sistemas de apoio técnico, logístico e de gestão buscam garantir a integralidade do cuidado*” ^18^. Quanto ao seu funcionamento em relação aos casos de aborto legal por gravidez decorrente de violência sexual, a impressão de alguns dos entrevistados foi de não a considerar efetiva em relação a esse tipo de assistência. Foi constatado que, nas experiências e percepções deles, há poucas instituições de saúde prestando esse tipo de intervenção, com justificativas que passam pela objeção de consciência à falta de material, entre outras.

Foram relatados encontros com pacientes vítimas de violência sexual na assistência que descrevem uma peregrinação por diversos serviços de saúde. Na fala de uma das entrevistadas, apareceu de forma mais explícita uma preocupação com mulheres vítimas de violência sexual que não conseguem efetivar o direito ao aborto legal de forma segura em uma instituição de saúde, seja por ter tido essa assistência negada, ou pela mulher não ter conseguido acessar o serviço para solicitar a seu direito, o que se relaciona ao território complexo do município onde a pesquisa foi realizada e às barreiras de acesso à saúde enfrentadas pela população de forma geral.

“*Apesar da lei existir, o sistema é muito falho* (...) *Muitas maternidades não fazem o aborto legal, mesmo isso sendo previsto em lei, e essas mulheres em situação de vulnerabilidade ficam completamente fora do sistema.* (...) *Se elas se convencem de realizar o aborto, elas vão ter que buscar outros métodos...*” (Participante 4).

Tais falas criam impressões sobre o acesso ao aborto legal que podem ser comprovadas pela literatura. Um estudo de Madeiro & Diniz ^19^ mapeou os serviços de aborto legal no Brasil, e identificou apenas 37 ativos durante o período em que a coleta de dados foi realizada. O estudo apontou, ainda, que não havia serviço ativo em 7 estados do país, e que em apenas 6 havia mais de 1 serviço. Foi encontrado, também, que 15 dos serviços ativos haviam realizado menos de 10 procedimentos nos últimos 10 anos, sendo que 4 desses serviços estavam localizados em capitais e eram os únicos serviços de aborto legal da região.

Um estudo mais recente ^20^, publicado em 2019, contatou 176 hospitais que constavam em listas do Sistema Único de Saúde (SUS) e/ou do Ministério da Saúde como provedores desse serviço. Desses, apenas 76 estabelecimentos estavam efetivamente realizando o aborto legal, sendo 35 deles localizados na Região Sudeste ^20^. Outra pesquisa ^21^ identificou 251 estabelecimentos de saúde que haviam registrado a realização de, pelo menos, um aborto por razões médicas e legais em 2019. Desses, 101 eram registrados como Serviços de Referência para Interrupção da Gravidez em Casos Previstos em Lei (SRIGCPL). Cabe pontuar que não é possível pressupor que o aborto legal é ofertado de forma sustentada e contínua nos demais estabelecimentos de saúde que não estavam registrados como SRIGCPL, bem como a impossibilidade de saber qual fora o motivo que levou à realização do procedimento, tendo em vista que, em tese, é vedado às instituições de saúde se negarem a realizar o aborto legal quando há risco iminente à vida da mulher. As autoras identificaram, ainda, 39 estabelecimentos de saúde registrados como SRIGCPL que não haviam realizado nenhum aborto legal naquele ano, e que mais da metade das mulheres em idade fértil do território brasileiro viviam em municípios em que não havia serviços de saúde que ofertavam a realização do aborto legal ^21^.

A preocupação na fala da médica entrevistada com mulheres vítimas de violência sexual em situação de maior vulnerabilidade que não conseguem efetivar seus diretos em relação ao aborto legal nos serviços de saúde também se ratifica pela literatura. A *Pesquisa Nacional de Aborto 2016* ^22^ aponta que o aborto é uma prática comum na vida das mulheres brasileiras, independentemente de faixa etária, classe social, nível educacional e grupos raciais. Entretanto, mulheres de baixa escolaridade e de raça/cor negra são as que mais morrem em decorrência dele, configurando-se enquanto uma iniquidade em saúde ^23^.

### As lacunas no percurso da formação profissional: a invisibilidade da temática

Quanto à percepção acerca da formação profissional, ficou evidente nas falas dos entrevistados que a temática é pouco abordada de forma geral nos diversos momentos do percurso de formação. Todos os entrevistados afirmaram ter tido um contato muito breve com o tema durante a graduação, e o ingresso na residência foi apresentado como o momento em que os casos de aborto legal e violência sexual efetivamente apareceram.

Para as residentes entrevistadas, o contato com o tema dentro da residência ocorre no dia a dia da assistência, e a presença do corpo médico da instituição, capacitado tecnicamente e que apresente uma conduta humanizada para com a paciente, foi destacada enquanto um diferencial no processo de ensino-aprendizagem. Entretanto, foi levantado um sentimento de falta em relação a espaços institucionalizados dentro da residência para discutir esses casos, bem como momentos formais de capacitação profissional. Essa fala vai ao encontro das respostas encontradas nos questionários, em que apenas 1 médico dos 36 participantes referiu ter participado de alguma prática educativa em relação ao tema na instituição da pesquisa.

Uma das residentes entrevistadas falou, também, que percebe essa falta em relação a outros espaços de formação, como em simpósios e congressos. Ela diz: 

“*Em congresso,* (...) *falando do aborto, não é tema, definitivamente não é… A única coisa que é, no sentido da violência* [sexual]*, é a prevenção de IST* [infecção sexualmente transmissível] *e gestação… Mas aborto, não… Definitivamente não é falado nesses eventos*” (Participante 5).

Os entrevistados que faziam parte do corpo médico da instituição abordaram a necessidade de se atualizarem em relação ao tema com o passar do tempo, destacando as diferenças no panorama do aborto legal no âmbito da saúde quando se tornaram médicos e atualmente, bem como os avanços dessas discussões na sociedade de forma geral.

Outro ponto levantado, ainda, foi o enfoque técnico da formação, não havendo muita atenção ao preparo em relação ao manejo do atendimento. Como explicitado nessa fala:

“*A paciente muitas vezes tá muito abalada psicologicamente… E é difícil pra gente ficar tocando no assunto que você sabe que abala mais a pessoa… A forma como você vai perguntar as informações necessárias… Você fica pensando antes de falar… Fica uma situação desconfortável, porque você não quer deixar o outro desconfortável.* (...) *Eu acho que a gente não tem esse preparo, na forma de perguntar, na forma de abordar.* (...) *Não é tão trabalhado isso no nosso dia a dia*” (Participante 1).

A escassez de contato com a temática durante a formação profissional também foi evidenciada pelo estudo de Farias & Cavalcanti ^12^. As autoras relacionam a falta de contato com o tema à acentuada dificuldade dos profissionais em lidarem com esses casos, o que interfere na promoção de uma assistência à saúde adequada a essas mulheres. Elas pontuam, ainda, o enfoque da visão técnico-curativa e biologista do processo saúde-doença na formação dos profissionais da saúde, especialmente dos médicos, o que resulta em um certo despreparo dos profissionais para lidarem com aspectos sociais, culturais e subjetivos relacionados ao cuidado à saúde.

Giugliani et al. ^3^ demonstram que a pouca discussão do tema no campo da saúde e da formação diz respeito à naturalização da violência contra a mulher no país, e mais especificamente sobre a invisibilidade da violência sexual, o que é reflexo de uma sociedade organizada segundo estruturas patriarcais. Quanto ao campo da saúde e da formação, percebe-se que o contexto histórico-cultural do país não costuma ser colocado seriamente em pauta. Assim, são poucos os espaços disponíveis nesse meio para que sejam discutidas questões como relações de gênero, desigualdades sociais, estruturas sociais e acesso aos direitos da população, o que leva a uma reprodução automática de estruturas estabelecidas.

### Ruídos entre o que é dito e o que é escutado: será que é verdade?

A desconfiança em relação à palavra da mulher quanto à veracidade do estupro apareceu de forma hegemônica na fala dos médicos do corpo da instituição, não tendo sido observada na fala das médicas residentes. Como explicitado nessa fala:

“*Nós moramos num país em que o aborto não é legalizado e aí por muitas vezes pacientes que decidem por fazer o aborto podem não ter passado pela situação de violência e usar um tipo de subterfúgio pra tentar burlar o sistema.* (...) *Como é uma questão subjetiva a gente fica muito na mão e aberto pra esse tipo de coisa acontecer… Então a gente tem que estar muito atento, tem que juntar o quebra cabeça, juntar a história, perceber realmente se existe veracidade ou não… Juntar a data da última menstruação.* (...) *E às vezes elas vêm tão treinadas...*” (Participante 2).

O sentimento de estar sendo enganado foi destacado por esses médicos. Um deles disse que esse sentimento acaba gerando barreiras para os profissionais e as instituições de saúde prestarem assistência ao aborto legal nesses casos. Ainda, repercussões do sentimento de desconfiança na assistência foram apresentadas por uma médica:

“*Às vezes também por a gente se sentir enganado em algumas situações… A gente já vai com muita desconfiança, por outras situações… E aí às vezes vira uma bola de neve… Desconta numa pessoa… Uma desconfiança… Mais perguntas do que deveria ou mais exames do que deveria… E a pessoa tá lá, ouvindo o bebê… A pessoa tá lá, visualizando o embrião… Ah, temos que conferir a idade gestacional, e mete na ultra, e aí mais um… Mais um martírio pra aquela pessoa que realmente teve o abuso, né…*” (Participante 1).

Resultados similares também podem ser encontrados em outros estudos, que identificaram nos profissionais envolvidos na prática do aborto legal um receio de estarem interrompendo uma gravidez que não fora fruto de um estupro, questionando-se sobre a possibilidade das mulheres estarem mentindo, o que expressa um descrédito em relação à palavra dessas ^7^ ^,^ ^24^.

Dios ^24^ disserta mais extensamente sobre a palavra da mulher e as práticas de produção de verdade nos serviços de aborto legal do Brasil. A autora entrevistou 82 profissionais de saúde que atuam em serviços de aborto legal em cinco capitais do país. O estudo encontrou falas similares entre si, referentes à suspeição da palavra da mulher e ao temor dos profissionais em serem confundidos com um serviço de aborto ilegal. Esses receios se relacionam diretamente à criminalização do aborto e falam sobre seus impactos mesmo na assistência a mulheres com direito ao aborto perante a legislação.

Dios ^24^ argumenta ainda que, nas outras situações em que o aborto é permitido no Brasil - não havendo outro meio de salvar a vida da gestante ou em casos de fetos anencéfalos -, as instituições de saúde se emparam em saberes biomédicos, como exames e laudos, por meio dos quais demarcam as fronteiras do que se enquadraria na lei. Por outro lado, nos casos de gravidez por estupro, não há saberes que demarquem esse limite. Nos textos da política pública, a palavra da mulher em relação ao estupro é tida como suficiente para o acesso ao aborto legal. Entretanto, ao se depararem com esses casos na assistência, os profissionais acabam agindo mediante uma política de suspeição, sendo a fala da mulher colocada constantemente sob avaliação por não haver outro meio de atestar a licitude do aborto.

Assim, a mulher que chega ao serviço de saúde em busca do aborto legal é vista inicialmente como alguém que pode estar mentindo, e não enquanto alguém em busca de um direito. Nessa política de suspeição, o serviço de aborto legal procura provas de verdade a respeito da violência sexual sofrida para além da palavra da mulher, por meio do que pode ser acessado pelo seu corpo, pela forma como se porta, por murmúrios e pelo que silencia ^24^.

Nesse jogo, o papel do profissional se confunde entre assistência à saúde e investigação e policiamento. Dios ^24^ cita Foucault para pensar na produção de verdade dentro desses serviços. Ao falar sobre a violência que sofreu, a mulher estaria confessando algo aos profissionais. Essa confissão, entretanto, se desenrola em uma relação de poder, em que o lado dominante é o de quem interroga e ouve. É a partir da escuta desse alguém que o discurso da vítima será legitimado como verdade. Entretanto, ele não será baseado apenas na história objetiva dos fatos, mas do que se espera socialmente de uma vítima de violência sexual. Nesse contexto, a vítima precisa narrar sua história de uma forma que convença o outro que ela tem direito ao aborto, apresentando sinais de trauma e de uma subjetividade específica do que se espera de quem foi vítima de violência sexual ^24^.

### O lugar de escuta: afetações de médicas obstetras

Foi possível perceber, na fala das médicas mulheres, uma maior afetação durante as entrevistas. Suas falas vieram mais carregadas de sentimentos e emoções, principalmente ao abordarem o encontro com mulheres vítimas de violência sexual na assistência, como evidenciado nessa fala:

“*Pra mim como mulher é muito… Eu lembro que algumas vezes eu falei isso pras pacientes… De, quando elas me contavam a história que tinha acontecido… De eu falar: ‘Cara, é… Eu sinto muito que você tenha que passar por isso. Eu fico toda arrepiada* [emocionada]*… Eu sinto muito que você tenha que passar por isso, porque, como mulher, isso dói em mim… Porque podia ser eu, sabe… Podia ser minha irmã, uma amiga, uma mãe, uma prima, sabe… Porque a gente sabe que, no mundo que a gente vive, é isso aí’*...” (Participante 4).

As entrevistadas mostraram uma maior sensibilização em relação à mulher em situação de violência, apresentando a dimensão de que a estrutura patriarcal também as afeta e as expõe a situações de violência, inclusive sexual. Esse reconhecimento repercute na assistência exercida por elas, as motivando a prestarem um atendimento mais humanizado, conforme preconizado pela Norma Técnica ^15^. Como destacado por uma entrevistada: 

“*Eu acho que, se eu fosse vítima de violência* (...)*, eu gostaria também de ter uma assistência que aceitasse a minha decisão e que me fosse tudo explicado e eu tivesse um bom atendimento*” (Participante 6).

Foi destacada, também, a forma como a cultura repercute na assistência prestada às mulheres vítimas de violência sexual que procuram assistência ao aborto legal. Foi citado o machismo e a estigmatização que elas sofrem nos serviços de saúde, atrelado ao controle dos corpos e da capacidade reprodutiva da mulher. Como explicitado nessa fala:

“*Eu vejo que tem muito preconceito com essas mulheres, muito machismo mesmo* (...) *Eu acho que isso teria que mudar muito… Mas não é só do atendimento, é mais uma questão de cultura, né… Cultural da população… Que apesar da gente ter muita informação sobre isso, o pessoal barra aí em uma questão pessoal e de preconceito mesmo com essas mulheres que resolvem não continuar com a gestação*” (Participante 6).

Dios ^24^ demonstra que pensar os serviços de aborto legal no Brasil por uma ótica feminista tem o poder de desafiar a inteligibilidade de gênero e a ordem patriarcal. Isso não significa desqualificar a assistência prestada pelos serviços, mas vislumbrar a possibilidade de abrir pequenas fissuras que nos permitam estranhar algumas práticas que nos são dadas como prontas. Quando entendemos que esse modelo que nos é dado como pronto pode ser sempre remodelado, abrimos espaços para novas possibilidades de produção de cuidado.

Pensando por meio de uma ética feminista, Diniz & Gebara ^25^ comprovam a importância de transformar o ato de ouvir em escutar. Elas explicam que o ato de ouvir pode ser passivo, irrefletido. O corpo e os afetos do ouvinte podem se manter intactos e indisponíveis perante as palavras enunciadas por quem fala. Em contrapartida, escutar envolve se deixar afetar pelas palavras e pelos silêncios do falante. Dessa forma, essa transformação nos auxiliaria a acessar as vivências de outros corpos, o que nos possibilitaria ir além de uma escuta engessada.

A prática da escuta pode ser incômoda para quem a exerce, pois ela nunca se completa e nos leva ao deslocamento das certezas. Ainda assim, aposta-se nela enquanto uma forma de acessar subjetividades de outros corpos ^25^. No contexto dos serviços de aborto legal, podemos entender que se oferecer em posição de escuta às mulheres vítimas de violência sexual compreende uma estratégia de humanização do cuidado, que visa se aproximar das vivências de mulheres muitas vezes estigmatizadas nos serviços de saúde.

## Considerações finais

O estudo teve como limitações o fato da maioria das entrevistas terem sido realizadas no ambiente médico, e o atravessamento da entrevistadora ser inserido no contexto institucional enquanto residente de psicologia.

Os resultados evidenciaram os dilemas enfrentados pelos profissionais na assistência ao aborto legal em casos de gravidez decorrente de violência sexual. Foi possível identificar a escassez de formação profissional em relação à temática, o que se relaciona à invisibilidade da violência contra a mulher no país e à estigmatização das vítimas de violência que procuram os serviços de saúde em busca do aborto legal.

A palavra da mulher foi tida ora como objeto de suspeição em relação à veracidade do estupro, ora como capaz de suscitar afetação das profissionais em suas escutas. Tal afetação possibilitou que essas profissionais se aproximassem das vítimas e oferecessem uma assistência mais humanizada, desafiando a ordem patriarcal de suspeição à palavra da mulher nesses casos.

A partir desses resultados, pensa-se na importância das temáticas do aborto legal e da violência sexual serem abordadas nos campos da saúde e da formação profissional para além do enfoque técnico-científico, visando produzir novas estratégias de cuidado.
